# BMPER alleviates ischemic brain injury by protecting neurons and inhibiting neuroinflammation via Smad3‐Akt‐Nrf2 pathway

**DOI:** 10.1111/cns.13782

**Published:** 2021-12-14

**Authors:** Peng Ding, Wei Chen, Xiaodi Yan, Jinxiang Zhang, Cheng Li, Guangming Zhang, Yongqiang Wang, Yonghua Li

**Affiliations:** ^1^ Department of Anesthesiology Changzheng Hospital Second Affiliated Hospital of Naval Medical University Shanghai China; ^2^ Department of Anesthesiology PLA 983 Hospital Tianjin China; ^3^ Department of Anesthesiology Tongren Hospital Shanghai Jiao Tong University School of Medicine Shanghai China; ^4^ Department of Anesthesiology & Research Institute for Acupuncture Anesthesia Shuguang Hospital Affiliated to Shanghai University of Traditional Chinese Medicine Shanghai China

**Keywords:** BMPER, cell death, ischemic stroke, neuroinflammation, Smad3

## Abstract

**Aims:**

Bone morphogenetic proteins (BMPs) are a group of proteins related to bone morphogenesis. BMP‐binding endothelial regulator (BMPER), a secreted protein that interacts with BMPs, is known to be involved in ischemic injuries. Here, we explored the effects of BMPER on cerebral ischemia and its mechanism of action.

**Methods:**

A mouse model of brain ischemia was induced by middle cerebral artery occlusion (MCAO). An in vitro ischemic model was established by subjecting primary cultured neurons to oxygen‐glucose deprivation/reperfusion (OGD/R). Serum levels of BMPs/BMPER were measured in MCAO mice and in patients with acute ischemic stroke (AIS). Brain damages were compared between BMPER‐ and vehicle‐treated mice. Quantitative polymerase chain reaction (qPCR), immunohistochemistry, and immunofluorescence staining were performed to examine neuroinflammation and cell death. BMPER‐related pathways were assessed by Western blotting.

**Results:**

BMPER level was elevated in MCAO mice and AIS patients. BMPER administration reduced mortality, infarct size, brain edema, and neurological deficit after MCAO. Neuroinflammation and cell death after ischemia were alleviated by BMPER both in vivo and in vitro. BMPER activated the Smad3/Akt/Nrf2 pathway in OGD/R‐challenged neurons.

**Conclusion:**

BMPER is a neuroprotective hormone that alleviates ischemic brain injury via activating the Smad3/Akt/Nrf2 pathway. These findings may provide potential therapeutic strategies for stroke.

## Graphical abstract

Terminal deoxynucleotidyl transferase dUTP nick‐end labeling (TUNEL) staining demonstrated that the number of dead/dying cells was pronouncedly increased in the brains of middle cerebral artery occlusion (MCAO) mice, which was attenuated by the bone morphogenetic protein‐binding endothelial regulator (BMPER) treatment.

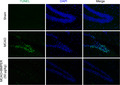

## 1 INTRODUCTION

Stroke is a leading cause of mortality and long‐term disability worldwide, resulting in substantial healthcare expenditures.^1^ About 80% of strokes are ischemic stroke, which is caused by occlusion of a large cerebral artery followed by cerebral blood supply interruption. In the past three decades, over 1000 drugs have failed to demonstrate benefits to stroke patients in clinical trials,^2^ suggesting that the pathology of ischemic stroke is far more complex than we previously thought. So far, intravenous thrombolysis and mechanical thrombectomy are the only effective approaches for acute ischemic stroke (AIS) patients with large vessel occlusion. However, the narrow therapeutic window of these therapies limits their application in stroke patients.^3^ Thus, it is imperative to explore the pathophysiological mechanism of ischemic stroke and seek new strategies for effective prevention/treatment.

Bone morphogenetic proteins (BMPs) are a group of signaling molecules that belong to the transforming growth factor‐β (TGF‐β) superfamily.^4^ These secreted cytokines are initially discovered as inducers of bone formation. They have later been recognized as multi‐functional regulators that are involved in a large variety of pathophysiological processes in almost all organs. Currently, about 15 structurally related BMPs have been identified.^4^ Some of them are associated with ischemic injuries, including myocardial ischemia and stroke. Kercheva et al. report that the serum levels of BMP2 and BMP4 increase in patients with acute myocardial infarction and decrease during the 6‐month follow‐up.^5^ In a rat model of brain ischemia induced by middle cerebral artery occlusion (MCAO), pretreatment with BMP6 improves motor function and reduces cerebral infarction without altering cerebral blood flow.^6^ BMP7 administration decreases body asymmetry and increases locomotor activity after MCAO.^7^ Moreover, BMP7 treatment promotes neuroregeneration in MCAO model through activating proliferation of neuronal precursors after ischemia.^8^

BMP‐binding endothelial regulator (BMPER), also known as crossveinless 2 (CV‐2), is a glycoprotein structurally associated with BMPs. It was cloned in 2003 as a secreted protein containing an amino‐terminal signal peptide, five cysteine‐rich domains, a von Willebrand D domain, and a trypsin inhibitor domain.^9^ BMPER can interact with BMP2, BMP4, and BMP6 directly, and antagonize BMP4‐dependent Smad5 activation and BMP4‐dependent endothelial cell differentiation.^9^ It was also reported that BMPER acts as a BMP agonist to promote endothelial cell sprouting and migration via sustaining Smad1/5 phosphorylation and ERK1/2 activation.^10^ During vascular endothelium development, BMPER preferentially binds and inhibits BMP9, thereby providing strong feedback inhibition on the BMP9/ALK1 signaling but not the BMP4/ALK2 signaling.^11^ The complex roles of BMPER in BMP signaling are further demonstrated in hematopoietic stem cells (HSCs). BMPER is associated with BMP signaling inhibition but is transcriptionally induced by BMP4, which enables the maturation of HSCs.^12^ Global and endothelial cell‐specific knockout of BMPER results in hyperinsulinemia, glucose intolerance, and insulin resistance in mice.^13^ Apart from the important role of BMPER in hematopoietic and vascular development, the influence of BMPER in other pathophysiological condition is poorly understood. Interestingly, Liu et al. reported that in response to myocardial ischemia, hepatocyte‐derived BMPER shows potent cardioprotection.^14^ These studies suggest that BMPER may have a variety of biological functions via a precise control of BMP activities.

The current study reveals changes in circulating BMPs/BMPER levels after MCAO in mice and in patients with AIS and evaluates the effect of BMPER treatment in the mouse MCAO model in vivo and in primary neuronal cultures subjected to oxygen‐glucose deprivation/reperfusion (OGD/R) in vitro. This study elucidates a protective role of BMPER in cerebral ischemia and improves our understanding of the pathophysiological functions of BMPER.

## 2 MATERIALS AND METHODS

### 2.1 Patient enrollment and blood sample preparation

Patients were recruited consecutively from January to December 2020 in Department of Neurology of Changzheng Hospital in Naval Medical University. Informed consents were obtained from all subjects, and all experiments were approved by the Ethics Committee of Biomedicine of Naval Medical University. AIS patients within 6 h from the onset of the brain ischemia symptoms and before thrombolytic therapy were recruited. AIS diagnosis was confirmed by clinical features and brain CT scan or MRI examination according to the Guidelines for the Early Management of Patients with AIS.^15^ The control subjects were recruited during the same period from physical examination center. Exclusion criteria included: under 18 or over 80 years old, pregnant, severe infection, severe liver or renal dysfunction, craniocerebral trauma, intracranial surgery, and malignant tumors. There was a total of 39 patients with AIS and 16 healthy volunteers. Blood samples were collected in coagulation‐promoting tubes. Tubes were fully agitated, and left at room temperature for 30 min. Serum was collected and stored at −80°C. Serum levels of BMPER were measured by an ELISA kit (ELH‐CV2‐1; RayBiotech) according to the manufacturer's instructions.

### 2.2 Animals and the mouse model of cerebral ischemia

Male C57BL/6 mice (8–12 weeks old) were purchased from Sino‐British Sippr/BK Lab Animal Ltd. Mice were housed in a facility with controlled temperature (23 ± 2℃) and lighting (08:00–20:00), with free access to food and tap water. All animal experiments were approved by the Ethics Committee of Biomedicine of Naval Medical University, performed in compliance with the National Institutes of Health Guide for Care and Use of Laboratory Animals, and reported in accordance with the Animal Research: Reporting In Vivo Experiments (ARRIVE) guidelines 2.0.^16^

Cerebral ischemia was induced by MCAO in mice as we previously described.^17^ Mice were anesthetized with pentobarbital (40 mg/kg, i.p.) and the core temperature (rectum) was maintained at 36.5–37.5°C using a homeothermic heating pad (CWE In.) throughout the surgery. Cerebral focal ischemia was produced by intraluminal occlusion of the left middle cerebral artery using a silicone rubber‐coated nylon monofilament. The cerebral blood flow was reduced by more than 85% as monitored by a laser Doppler flowmeter (VMS^TM^‐LDF1; Moor Instruments). Two hours after MCAO, the occluding filament was withdrawn to allow reperfusion.

### 2.3 Drug administration and sample harvest

Mice were injected through tail vein with recombinant BMPER (2299‐CV‐050; R&D Systems) dissolved in saline 5 min post‐MCAO. A total of 54 mice were used for survival analysis for 2 weeks after surgery. The mice were divided into five groups: Sham (*n* = 6), MCAO (*n* = 12), MCAO+BMPER (5 μg/kg, *n* = 12), MCAO+BMPER (50 μg/kg, *n* = 12), and MCAO+BMPER (100 μg/kg, *n* = 12). Survival analysis was conducted by Kaplan–Meier survival curve.

The effect of BMPER on acute brain ischemia was assessed using another batch of mice. Three groups of mice (Sham, *n* = 8; MCAO, *n* = 10; MCAO+BMPER [50 μg/kg, *n* = 10]) were subjected to neurological deficit assessment at 24 h post‐MCAO and then sacrificed. Blood was collected into Eppendorf tubes and left standing at room temperature for 2 h. Serum was collected by centrifuge for 20 min at 3000 *g* and was stored at −80°C until use. For brain tissue sampling, the non‐ischemic tissues and infarcted tissues were dissected carefully and kept frozen at −80°C until use. The ischemic and non‐ischemic areas were distinguished by color and texture. The ischemic core was visibly loose and pale, while the non‐ischemic area was plump and shiny. The area between ischemic core and non‐ischemic area was penumbra, which was defined as a hypoperfused, metabolically active region surrounding the ischemic core.^18^

### 2.4 Neurological deficit assessment

The mice were examined for neurological deficit using the Bederson scoring^19^ and Garcia scoring.^20^ For Bederson scoring, mice with normal motor function were scored as 0, flexion of the contralateral torso and forearm upon lifting by the tail as 1, circling to the contralateral side but normal posture at rest as 2, leaning to the contralateral side as 3, and no spontaneous motor activity as 4. The modified Garcia scoring system consisted of six tests (spontaneous activity, symmetry in the movement of four limbs, forepaw outstretching, climbing, body proprioception, and response to vibrissae touch) with a maximal score of 18, and with higher scores indicating better performance.

### 2.5 Brain water content

The wet‐to‐dry weight ratio was used to determine the brain water content, which is an index of brain edema after MCAO‐induced cerebral ischemia.^21^ The method was modified based on our previous experience.^22^ Briefly, after euthanizing the mice, brains were quickly divided into cortex and striatum and weighed immediately. The samples were dried in an oven at 90°C for 24 h. The dried samples were reweighed, and brain water content was calculated as ([wet weight − dry weight]/wet weight) × 100%.

### 2.6 2,3,5‐triphenyl‐2h‐tetrazolium chloride staining

The brain was removed quickly and cut into slices with brain‐cutting matrix (ASI Instruments, USA). The slices were bathed in the 2,3,5‐triphenyl‐2h‐tetrazolium chloride (TTC) (Sigma) solution at 37°C for 30 min and then photographed. Infarction volume was the sum of all lesion areas multiplied by slice thickness and calculated using the Image J software (National Institutes of Health).^19^

### 2.7 Primary neuronal culture

The dissociated cortical cells were added to poly‐L‐lysine‐coated culture plates and maintained in Neurobasal^TM^ medium (Gibco) with 2% B27 supplement.^23^ Glial growth was suppressed by addition of 5‐fluoro‐2‐deoxyuridine. The purity of neuronal culture was >95%. The neurons were used for experiments after 6 days in vitro. BMPER was added into the culture medium to achieve the final concentrations of 1, 10, and 100 ng/ml.

### 2.8 Oxygen and glucose deprivation/reperfusion

OGD/R was performed as described previously.^24^ The primary neurons at a density of 1 × 10^5^ cells per well were cultured in a pre‐heated RPMI‐1640 glucose‐free medium (Gibco). The plate was put into an anaerobic culture bag (MITSUBISHI Gas Chemical), which included an anaerobic gas‐producing bag and an anaerobic indicator. The anaerobic culture bags containing six‐well plates were put into a CO_2_ incubator for 2, 4, or 6 h. The anaerobic devices were then removed, and the cells were cultured in a full culture medium at 37°C with 5% CO_2_ for 24 h.

### 2.9 Real‐time quantitative PCR

Real‐time quantitative PCR was performed as described previously.^25^ Total RNA was isolated from brain tissues using RNAiso Reagent (9108; TaKaRa) and reverse transcripted into cDNA using PrimeScript RT‐PCR kit (RR037A; TaKaRa) according to the manufacturer's instruction. Primers were designed using the Primer Express software version 1.5 (Applied Biosystems) and the sequences are listed in Table 1. The reaction of qPCR was performed using ABI Prism 7500 (Applied Biosystems) with TB Green^®^ Premix Ex Taq^TM^ (RR820A; TaKaRa). The housekeeping gene GAPDH was used as an internal control, and the relative gene expression was calculated using the 2^−ΔΔCt^ method.

**TABLE 1:** Primers used for real‐time quantitative PCR

Gene	Forward (5′–3′)	Reverse (5′–3′)
GAPDH	TCTGACGTGCCGCCTGGAG	TCGCAGGAGACAACCTGGTC
BMPER	CGCTCGCCTGGGATTA	CCCTTCATTTTCACATTTTGC
TNF‐α	TTCTGTCTACTGAACTTCGGGGTGATCGGTCC	GTATGAGATAGCAAATCGGCTGACGGTGTGGG
IL‐1β	GAAATGCCACCTTTTGACAGTG	TGGATGCTCTCATCAGGACAG
IL‐6	ATGGATGCTACCAAACTGGAT	TGAAGGACTCTGGCTTTGTCT

### 2.10 Western blotting

Western blotting was performed as described previously.^26^ The brain tissues or cells were homogenized in RIPA lysis buffer (P0013; Beyotime) with protease/phosphatase inhibitor cocktail (P0044/P8340; Sigma Aldrich). Lysates were centrifuged at 14,000 *g* for 30 min at 4°C. Total protein concentrations were measured using the Bradford assay kit (P0006; Beyotime). Supernatants were mixed with an equal volume of 2× loading buffer (Santa Cruz) and boiled for 10 min. Protein samples were electrophoresed and separated in a 12% sodium dodecyl sulfate‐polyacrylamide gel electrophoresis (SDS‐PAGE) gel and transferred onto a 0.45 μm nitrocellulose membranes at 100 V for 1 h. After blocking by 5% nonfat milk, the membrane was incubated over night at 4°C with the following primary antibodies: p‐Akt (2965; CST), t‐Akt (9272; CST), p‐Smad1 (9516; CST), p‐Smad2 (3104; CST), p‐Smad3 (9520; CST), Bcl‐2 (bs‐4563R; Bioss), Bax (bs‐4564R; Bioss), p‐Nrf2 (ab76026; Abcam), Nrf2 (ab76026; Abcam), tubulin (sc‐8035; Santa Cruz), or β‐actin (sc‐1265; Santa Cruz). Membrane was washed with Tris Buffered Saline with Tween 20 (TBST) three times and incubated with horseradish peroxidase‐labeled secondary antibody for 1 h at room temperature. The immunoblotting was detected using an enhanced chemiluminescence detection kit (WBKLS0500; Millipore). Grayscale analysis was performed using the ImageJ software.

### 2.11 Immunohistochemistry

Immunohistochemistry was performed as described previously.^17^ The primary antibodies used were as follows: TNF‐α (ab183218; Abcam), IL‐6 (ab214429; Abcam), IL‐1β (sc‐52012; Santa Cruz), Iba‐1 (sc‐32725; Santa Cruz), ICAM‐1 (ab119871; Abcam), NOX1 (bs‐3682R; Bioss), and NOX4 (bs‐1091R; Bioss). The images were analyzed according to the immunoreactivity intensity (brown) using the Image J software. All procedures were carried out in a double‐blinded manner.

### 2.12 Immunofluorescent staining

The cells cultured on slides were fixed for 30 min in ice‐cold 4% paraformaldehyde. After being washed with PBS for three times, the slides were incubated with 5% normal goat serum followed by primary antibodies against HuD (dilution 1:500, sc‐48421; Santa Cruz) overnight at 4°C. After being washed with PBS, the slides were incubated with Cy3‐labeled secondary antibody (dilution: 1:100) in a dark chamber. The slide was incubated with 4′,6‐diamidino‐2‐phenylindole (DAPI) for 5 min and then coverslipped with ProLong™ Gold Antifade Mountant (Thermo). The immunofluorescence was captured using the IX‐71 fluorescent scanning microscope (Olympus). The number of HuD^+^ cells was quantitated for the survival neurons after OGD/R.

### 2.13 TUNEL assay

The immunofluorescent terminal deoxynucleotidyl transferase dUTP nick‐end labeling (TUNEL) assay was performed with DeadEnd™ Fluorometric TUNEL kit (Promega) as described previously.^27^, ^28^ Images were acquired by a fluorescence microscope (IX‐71) with a digital camera. The percentage of cell death was calculated as the total number of TUNEL‐positive nuclei (green) divided by the total number of DAPI‐positive nuclei (blue). Images were captured in the ischemic area and quantitatively assessed using the ImageJ software.

### 2.14 Cell viability and ROS evaluation

Cell viability was determined using a commercial CCK‐8 kit (C0037; Beyotime) according to the manufacturer's instruction. ROS evaluation was performed using a dihydroethidium (DHE)‐based method as described previously.^29^ Malondialdehyde (MDA) was determined using a commercial kit (S0131; Beyotime) according to the manufacturer's instruction.

### 2.15 Activities of Caspase‐3, ‐8, and ‐9

Activities of Caspase‐3, ‐8, and ‐9 were assessed using commercial kits (C1116, C1152, C1158; Beyotime) according to the manufacturer's instructions. Briefly, brain tissues were lysed and homogenized. The supernatants of the homogenates were harvested by centrifugation at 12,000 *g*, and the protein concentration was determined using a bicinchoninic acid (BCA) protein assay kit (P0012; Beyotime). The lysates were incubated with Ac‐DEVD‐pNA (2 mmol/L, for Caspase‐3 activity), Ac‐IETD‐pNA (2 mmol/L, for Caspase‐8 activity), and Ac‐LEHD‐pNA (2 mmol/L, for Caspase‐9 activity) at 37°C for 2 h. After incubation, absorbance was read at 405 nm using a microplate reader (BioTek). Fold‐increases in activities of Caspase‐3, ‐8, and ‐9 activities were determined by comparison with control (non‐ischemic brain tissue).

### 2.16 Statistical analysis

Statistical analyses were conducted using the GraphPad Prism 9.0 (GraphPad Software) and SPSS 25.0 (IBM Corporation). Data normality was assessed by the Shapiro–Wilk test. Continuous variables with normal distributions were described as mean ± SEM, and the intergroup differences were analyzed by Student's *t*‐test (two groups) or one‐way ANOVA followed by LSD post hoc test (≥3 groups). Non‐normally distributed data were described as median (Q1–Q3) and analyzed by Mann–Whitney *U*‐test (two groups). The differences in means among multiple groups of non‐parametric data were analyzed by the Kruskal–Wallis test. A *p*‐value less than 0.05 was considered statistically significant.

## 3 RESULTS

### 3.1 Serum BMPER is induced upon cerebral ischemia in mouse and human

**FIGURE 1:**
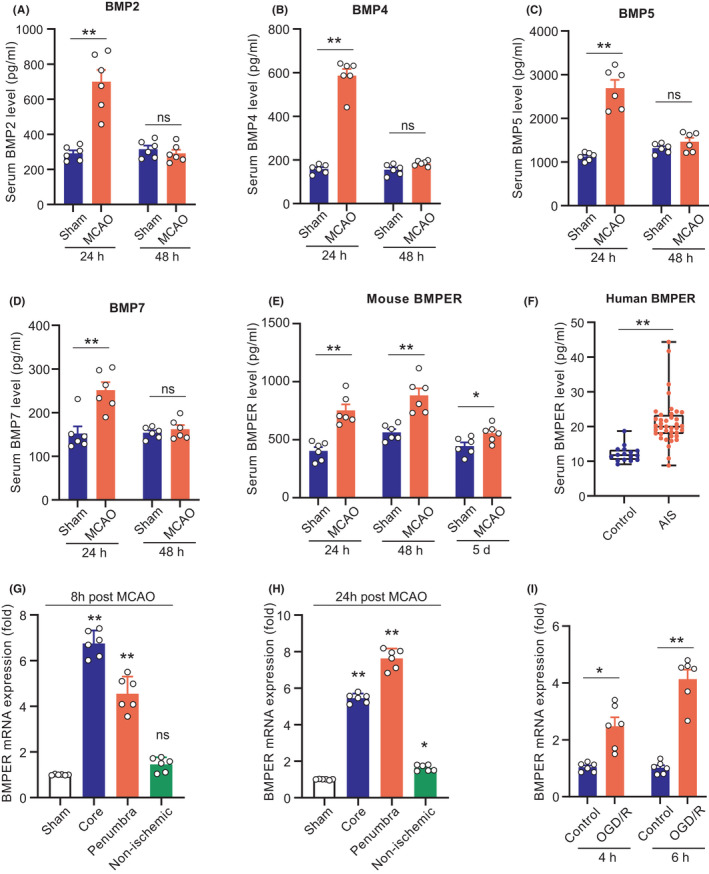
Serum BMPER is induced upon cerebral ischemia in mouse and human. (A–E) Serum levels of BMP2, BMP4, BMP5, BMP7, and BMPER at 24 and 48 h post‐MCAO. Data are expressed as mean ± SEM, *n* = 6, *p < 0.05, **p < 0.01 by Student's *t*‐test. (F) Serum BMPER levels in 39 AIS patients and 16 healthy volunteers. The blood was drawn within 6 h from the onset of the brain ischemia symptoms and before thrombolytic therapy. Data are expressed as median (Q1–Q3, range), **p < 0.01 by Mann–Whitney *U*‐test. (G, H) The mRNA expression of BMPER in ischemic core, penumbra, and non‐ischemic area in MCAO mice and sham‐operated mice. Data are expressed as mean ± SEM, *n* = 6, *p < 0.05, **p < 0.01 versus Sham by ANOVA followed by LSD‐t post hoc test. (I) The mRNA expression of BMPER in primary cultured neurons under OGD/R stress for 4 and 6 h. Data are expressed as mean ± SEM, *n* = 6, **p < 0.01 by Student's *t*‐test. AIS, acute ischemic stroke; BMP, bone morphogenetic protein; BMPER, BMP‐binding endothelial regulator; MCAO, middle cerebral artery occlusion; OGD/R, oxygen‐glucose deprivation/reperfusion

To screen which BMP is extensively induced by cerebral ischemic stress, we measured the serum levels of several members in the BMP family, including BMP2, BMP4, BMP5, BMP7, and BMPER in MCAO mice. We found that BMP2 significantly increased 24 h post‐MCAO and returned to baseline at 48 h (Figure 1A). Similar changes were observed in BMP4 (Figure 1B), BMP5 (Figure 1C), and BMP7 (Figure 1D). The serum BMPER levels significantly increased at both 24 and 48 h post‐MCAO (Figure 1E). And remained elevated 5 days post‐MCAO (Figure 1E). These data suggest a prolonged involvement of BMPER in the pathophysiology of brain ischemia, while the changes in the serum levels of BMP2, BMP4, BMP5, and BMP7 might be acute phase reactions.

We further confirmed the changes in serum BMPER levels in samples collected from 39 patients with AIS and 16 control healthy volunteers. The serum BMPER levels in AIS patients were significantly higher than that in control subjects (19.9[17.9–23.4] ng/ml vs. 11.7[10.4–13.3] ng/ml, *p* < 0.01; Figure 1F).

We also evaluated the changes in brain BMPER levels upon cerebral ischemia. Brain tissues were collected from three different areas (ischemic core, penumbra, and non‐ischemic area) 8 and 24 h after MCAO. Normal brain tissues collected from the sham‐operated mice were used as controls. RT‐PCR was used to determine the mRNA levels of BMPER. At 8 h post‐MCAO, the BMPER levels were about 6‐fold and 3.5‐fold higher in the ischemic core and penumbra, respectively, compared with that in sham brain tissue (Figure 1G). There was no difference in BMPER levels between the non‐ischemic tissues in MCAO mice and the sham brain tissue. At 24 h post‐MCAO, the BMPER level remained elevated (~8‐fold) in the penumbra area (Figure 1H) and slightly but significantly increased in the non‐ischemic tissues in MCAO mice compared with the normal brain tissue from sham‐operated mice. It seems that the BMPER mRNA expression is extensively induced in the brain (even in the non‐ischemic area) by ischemic stress.

We further investigated the influence of OGD/R on BMPER mRNA levels in primary mouse neurons. The BMPER mRNA levels increased to approximately 3‐fold of normal levels at 4 h post‐OGD/R (Figure 1I). This upregulation of BMPER was more pronounced at 6 h post‐OGD/R (Figure 1I).

Taken together, our in vivo and in vitro results consistently demonstrate upregulations of BMPER upon ischemic stress.

### 3.2 BMPER ameliorates brain injury after MCAO

**FIGURE 2:**
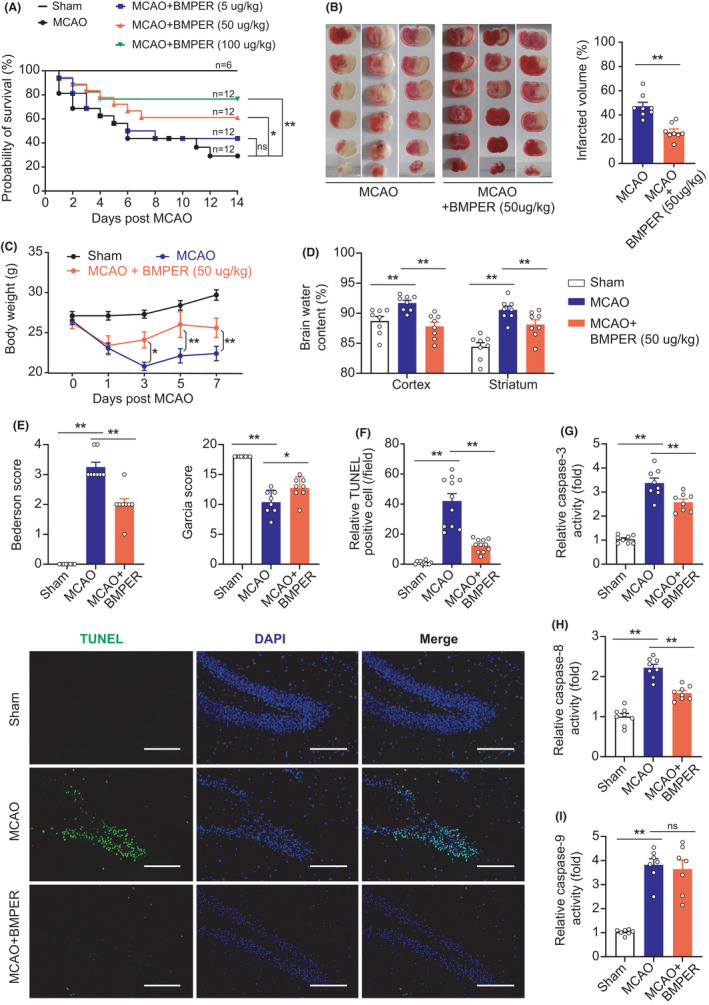
BMPER ameliorates brain injury and decreases cell death after MCAO. (A)The Kaplan‐Meier survival curve of MCAO mice receiving different doses of recombinant BMPER (5, 50 and 100 μg/kg). *p<0.05, **p < 0.01 by log‐rank test. NS, no significance. (B) TTC staining showed the brain infarct area of MCAO mice receiving recombinant BMPER (50 μg/kg). (C) Body weight of MCAO mice receiving recombinant BMPER. Data in B&C are expressed as mean ± SEM, *n* = 8, *p < 0.05, **p < 0.01 by Student's *t*‐test. (D) Brain water content in mice receiving recombinant BMPER (50 μg/kg). Data are expressed as mean ± SEM, *n* = 8. **p < 0.01 by ANOVA followed by LSD‐t post hoc test. (E) Neurological deficit evaluation in mice receiving recombinant BMPER (50 μg/kg) using Bederson scoring and Garcia scoring. Data are expressed as mean ± SEM, *n* = 8. *p < 0.05, **p < 0.01 by Kruskal–Wallis test. (F) TUNEL assay showing the cell death in penumbra area of MCAO mice receiving BMPER treatment. Data are expressed as mean ± SEM, *n* = 11. **p < 0.01 by ANOVA followed by LSD‐t post hoc test. Scale bar = 100 μm. (G–I) The activities of caspase‐3, caspase‐8, and caspase‐9 in penumbra area of MCAO mice treated by recombinant BMPER. Data are expressed as mean ± SEM, *n* = 8. **p < 0.01 by ANOVA followed by LSD‐t post hoc test. BMP, bone morphogenetic protein; BMPER, BMP‐binding endothelial regulator; DAPI, 4′,6‐diamidino‐2‐phenylindole; MCAO, middle cerebral artery occlusion; NS, no significance; TUNEL, terminal deoxynucleotidyl transferase dUTP nick‐end labeling

Since both systemic and brain BMPER are upregulated by cerebral ischemia, we proposed that the upregulated BMPER has a critical role in pathophysiology of brain ischemia. We injected recombinant BMPER (5, 50, and 100 μg/kg body weight) through tail vein into MCAO mice. BMPER administration at the dose of 5 μg/kg had no effect on the survival of MCAO mice. However, BMPER administration at the dose of 50 and 100 μg/kg significantly decreased the mortality of MCAO mice (Figure 2A). The dose of 50 μg/kg was used as the optimal dose in the following experiments. TTC staining demonstrated that BMPER significantly reduced brain infarct area in MCAO mice (from 42% to 25%, *p* < 0.01) (Figure 2B). BMPER also ameliorated the body weight loss in MCAO mice (Figure 2C). In addition, BMPER significantly reduced the brain water content in the cortex and striatum of MCAO mice (Figure 2D), suggesting that BMPER ameliorated brain edema after ischemia. Both Bederson scoring and Garcia scoring showed that BMPER treatment reduced the neurological deficits caused by brain ischemia (Figure 2E).

### 3.3 BMPER decreases cell death after cerebral ischemic injury

Next, we evaluated the cell death in brain tissue at 24 h post‐MCAO. TUNEL staining demonstrated that the number of dead/dying cells was pronouncedly increased in the brains of MCAO mice, which was attenuated by the BMPER treatment (Figure 2F). In support of this, we found that the induced activities of caspase‐3 (Figure 2G) and caspase‐8 (Figure 2H) upon brain ischemia were partially blocked by BMPER. Interestingly, BMPER failed to suppress caspase‐9 activity (Figure 2I). These results suggest that BMPER decreases extrinsic apoptosis (caspase‐8 dependent) after cerebral ischemia.

### 3.4 BMPER reduces post‐ischemic neuroinflammation

**FIGURE 3:**
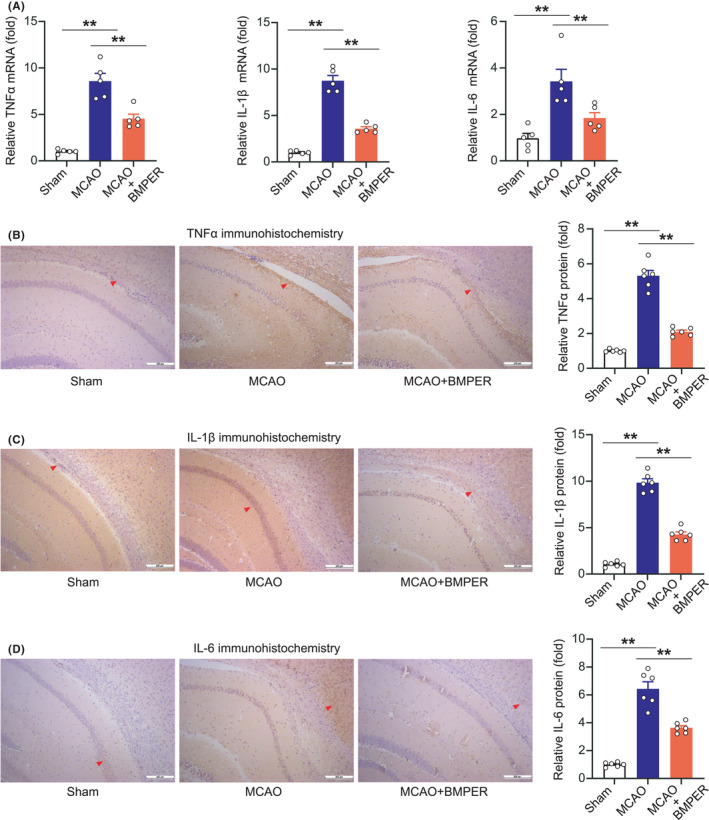
BMPER reduces pro‐inflammatory factors in MCAO model. (A) Relative mRNA levels of TNF‐α, IL‐1β, and IL‐6 in penumbra area of MCAO mice receiving BMPER treatment. Data are expressed as mean ± SEM, *n* = 5. **p < 0.01 by ANOVA followed by LSD‐t post hoc test. (B–D) Immunohistochemistry staining of TNF‐α, IL‐1β, and IL‐6 in penumbra area of MCAO mice receiving BMPER treatment. Data are expressed as mean ± SEM, *n* = 6. **p < 0.01 by ANOVA followed by LSD‐t post hoc test. BMP, bone morphogenetic protein; BMPER, BMP‐binding endothelial regulator; MCAO, middle cerebral artery occlusion

To monitor post‐ischemic neuroinflammation, we examined the mRNA and protein levels of pro‐inflammatory factors (TNF‐α, IL‐1β, and IL‐6) in mouse brain tissue at 24 h post‐MCAO. Brain ischemia significantly upregulated mRNA expression of TNF‐α, IL‐1β, and IL‐6, which were inhibited by BMPER (Figure 3A). We further performed immunohistochemistry assays to confirm these changes. TNF‐α, IL‐1β, and IL‐6 protein levels were indeed upregulated after MCAO, which were significantly suppressed by BMPER (Figure 3B–D). These data suggest that BMPER reduces pro‐inflammatory factors in brain tissue after ischemia.

**FIGURE 4:**
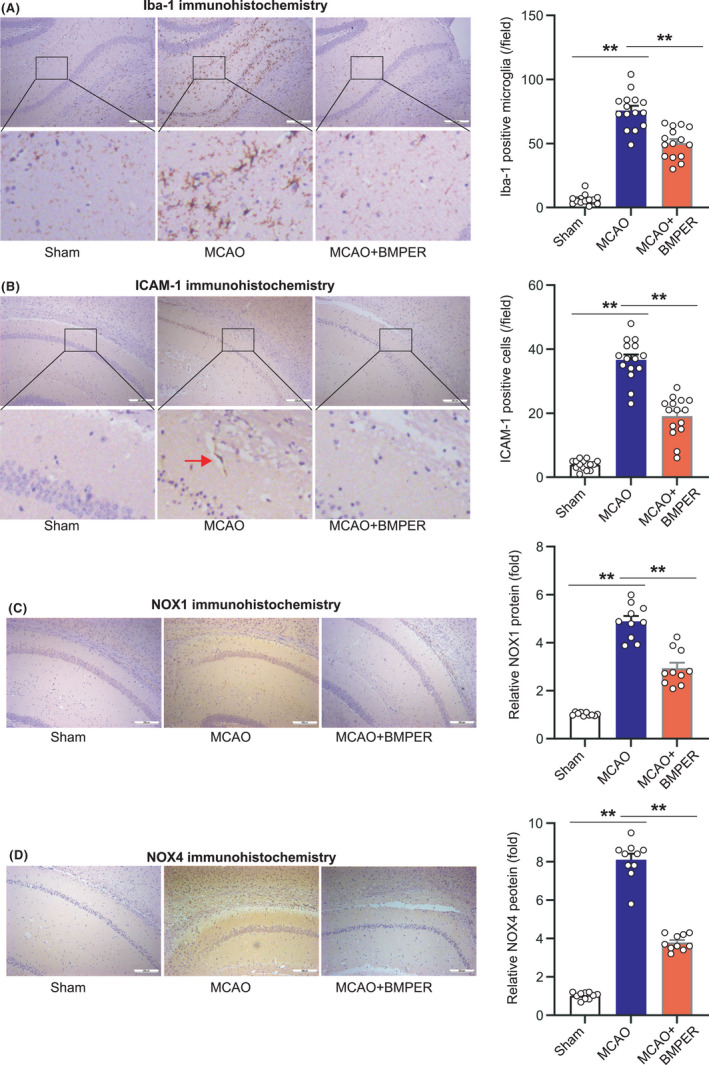
BMPER inhibits microglial activation, adhesion molecule, and superoxide‐generating enzyme in MCAO model. (A) Immunohistochemistry staining of microglia activation marker Iba‐1 in penumbra area of MCAO mice receiving BMPER treatment. (B) Immunohistochemistry staining of adhesion molecule ICAM‐1 in penumbra area of MCAO mice receiving BMPER treatment. (C, D) Immunohistochemistry staining of superoxide‐generating enzymes NOX1 and NOX4 in penumbra area of MCAO mice receiving BMPER treatment. Data are expressed as mean ± SEM, *n* = 6. **p < 0.01 by ANOVA followed by LSD‐t post hoc test. BMP, bone morphogenetic protein; BMPER, BMP‐binding endothelial regulator; MCAO, middle cerebral artery occlusion

We then evaluated ischemia‐induced immune cell activation and infiltration. Iba‐1, a marker of microglia, was determined using immunohistochemistry analysis. In brain tissue of sham‐operated mice, there were very few Iba‐1‐positive resting microglia with thin processes (Figure 4A). Classical morphology of activated microglia, such as hypertrophied and reduced branching, was detected in MCAO mice. BMPER substantially reduced the number of activated microglia (Figure 4A). ICAM‐1, a glycoprotein expressed on activated endothelial cells, was scarcely observed in brain sections of sham‐operated mice (Figure 4B). In contrast, the ICAM‐1 positive endothelial cells were noted (Figure 4B, red arrow) after MCAO. BMPER decreased the number of ICAM‐1‐positive endothelial cells (Figure 4B).

Finally, we evaluated protein levels of NOX1 and NOX4, the markers of oxidative stress. The diffusely expressed NOX1 and NOX4 in MCAO mice were significantly suppressed by BMPER (Figure 4C,D).

All these findings indicate that BMPER treatment reduces post‐ischemia neuroinflammation after ischemic stroke.

### 3.5 BMPER protects neuronal survival in the OGD/R model

**FIGURE 5:**
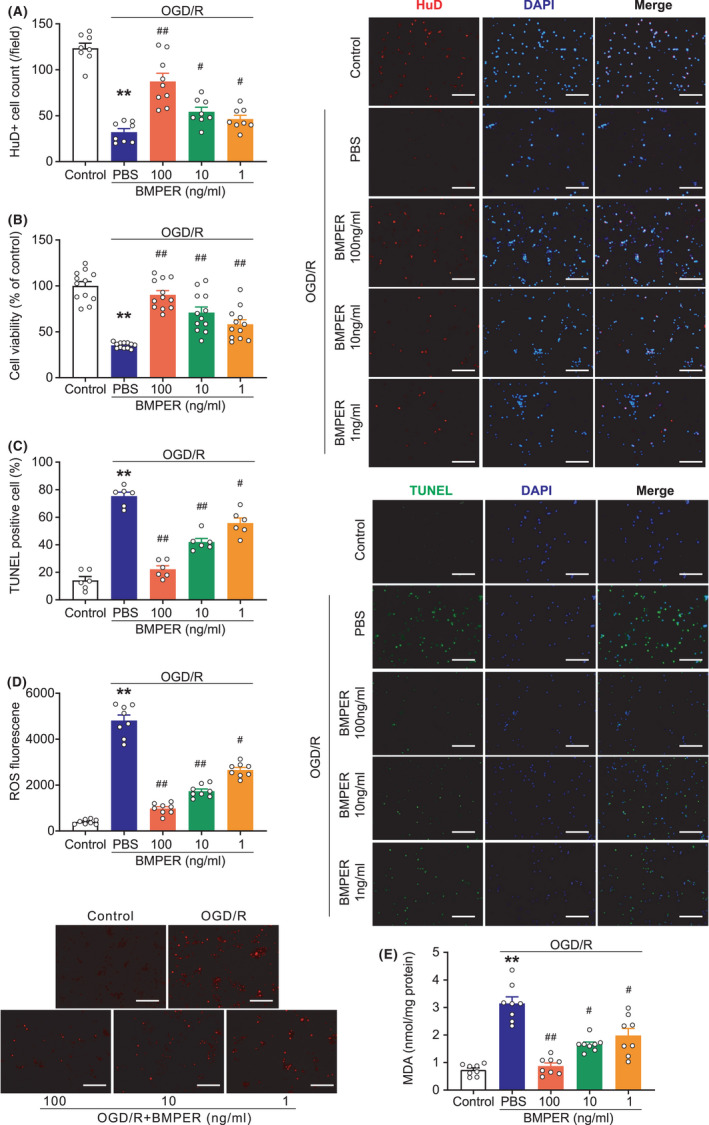
BMPER protects neuronal survival in the OGD/R model. (A) Immunofluorescence staining of live neurons using antibody against HuD, a neuronal marker. DAPI was used to stain nuclei. *n* = 8. (B) Cell viability of primary neurons subjected to OGD/R stress. *n* = 12. (C) Immunofluorescence TUNEL showing apoptosis in primary neurons subjected with OGD/R stress. *n* = 6. (D) ROS production determined by DHE immunofluorescence staining. *n* = 8. (E) Lipid oxidative stress was evaluated by MDA content. *n* = 8. Data are expressed as mean ± SEM. **p < 0.01 versus control; ^#^ *p* < 0.05, ^##^ *p* < 0.01 versus PBS by ANOVA followed by LSD‐t post hoc test. Scale bar = 100 μm. BMP, bone morphogenetic protein; BMPER, BMP‐binding endothelial regulator; DAPI, 4′,6‐diamidino‐2‐phenylindole; DHE, dihydroethidium; MDA, malondialdehyde; OGD/R, oxygen‐glucose deprivation/reperfusion; PBS, phosphate buffered saline; ROS, reactive oxygen species

To ascertain the neuroprotective impact of BMPER against brain ischemia, we tested the effect of recombinant BMPER on primary mouse neurons in an OGD/R model. HuD is a neuronal‐specific RNA‐binding protein.^30^ Immunofluorescent staining of HuD showed that OGD/R caused an obvious decline in the number of neurons, which was mitigated by BMPER supplement (1, 10, and 100 ng/ml) in a dose‐dependent manner (Figure 5A). Cell viability assay further confirmed this result (Figure 5B). TUNEL analysis showed that approximately 80% neurons underwent cell death in OGD/R condition, while BMPER significantly blocked the OGD/R‐induced cell death in a dose‐dependent manner (Figure 5C). ROS production assessed by DHE immunofluorescent staining demonstrated that BMPER treatment inhibited OGD/R‐induced ROS production (Figure 5D). BMPER supplement reduced the content of MDA, a marker of lipid peroxidation (Figure 5E). All these data indicate that BMPER enhances neuronal survival in an OGD/R model.

### 3.6 BMPER regulates Bcl‐2/Bax ratio and activates Smad3/Akt/Nrf2 signaling pathway

**FIGURE 6:**
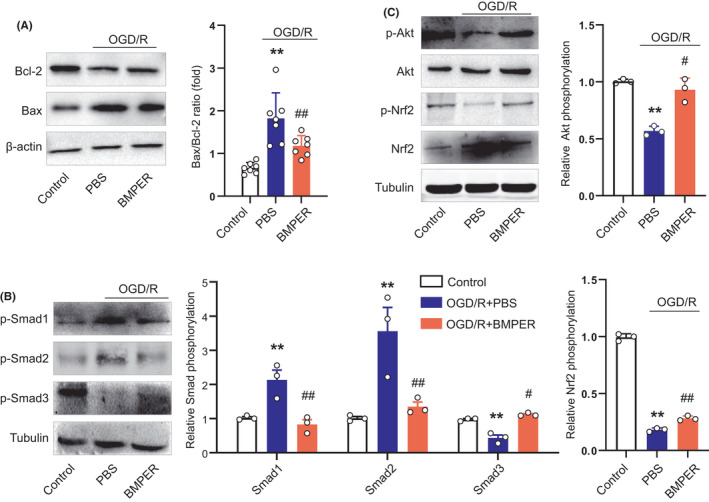
BMPER regulates Bcl‐2/Bax ratio and activates Smad3/Akt/Nrf2 signaling pathway. (A) Effects of BMPER on Bcl‐2 and Bax expression in neuron OGD/R model. *n* = 6. (B) Effects of BMPER on phosphorylated Smad1, Smad2, and Smad3 expression in neuron OGD/R model. *n* = 3. (C) Effects of BMPER on phosphorylated Akt and Nrf2 expression in neuron OGD/R model. *n* = 3. Data are expressed as mean ± SEM. **p < 0.01 versus control. ^#^ *p* < 0.05, ^##^ *p* < 0.01 versus PBS by ANOVA followed by LSD‐t post hoc test. BMP, bone morphogenetic protein; BMPER, BMP‐binding endothelial regulator; OGD/R, oxygen‐glucose deprivation/reperfusion; PBS, phosphate buffered saline

Next, we explored the molecular mechanisms underlying the neuroprotection by BMPER. OGD/R resulted in a marked decrease of Bcl‐2/Bax ratio in primary neurons, which was prevented by BMPER treatment (Figure 6A). It has been reported that BMPER contributes to the precise control of BMPs activities in different pathophysiological conditions.^10^, ^12^, ^31^ We therefore examined the influence of BMPER on the phosphorylation of Smad family members, which are the major transcription factors mediating the biological action of BMPs.^32^ We found that p‐Smad1 and p‐Smad2 were upregulated, whereas p‐Smad3 was downregulated by OGD/R (Figure 6B). BMPER treatment substantially reversed these changes. Moreover, OGD/R significantly decreased the phosphorylation of Akt and Nrf2, which were partly reversed by BMPER (Figure 6C). These data suggest that BMPER regulates Bcl‐2/Bax ratio and activates Smad3/Akt/Nrf2 signaling pathway upon cerebral ischemic stress.

## 4 DISCUSSION

In the present study, we provide the first evidence that BMPER ameliorates cerebral ischemia injury in vivo and in vitro. The first interesting finding of this study is that BMPER is induced by ischemic insult in both mouse model and clinical patients. Our screen experiment showed that most BMP family members, including BMP2, BMP4, BMP5, and BMP7 increased transiently at 24 h after brain ischemia. Unlike these BMPs, circulating BMPER levels remained elevated until subacute phase after brain ischemia. Additionally, BMPER mRNA expression was induced in penumbra area of MCAO brains and in primary neurons subjected to OGD/R insult. We further confirmed in AIS patients that serum BMPER levels were significantly increased after stroke. All these results highlight a critical role of BMPER in pathophysiology of brain ischemia.

We further identified BMPER as a neuroprotective molecule against ischemic brain injury. Of potential translational significance, the delivery of recombinant BMPER via tail vein significantly prolonged survival of MCAO mice in a dose‐dependent manner. BMPER treatment reduced brain infarct volume, weight loss and neurological deficit. Moreover, BMPER decreased ischemic cell death, evidenced by reduced number of TUNEL‐positive neural cell in the ischemic brain. It should be noted that BMPER treatment decreased caspase‐3/8 activities but showed no effect on caspase‐9 activity. As one of the intensively studied caspases, caspase‐9 is the initiator of intrinsic apoptosis. It has been well established that Apaf‐1 forms oligomers and activates pro‐caspase‐9 in a cytochrome C‐dependent pathway, initiating a caspase cascade involving the downstream executioner caspase‐3, ‐6, and ‐7.^33^ This capase‐9‐mediated intrinsic apoptosis pathway is independent of death receptor and caspase‐8,^34^ which act as initiators of extrinsic apoptosis.^35^ Therefore, we consider that BMPER may control extrinsic apoptosis but not intrinsic apoptosis in ischemic stroke. Lagna et al. show that BMPs promote apoptosis in pulmonary artery smooth muscle cells via activating caspases‐3, ‐8, and ‐9.^36^ The specific inhibition of BMPER on caspase‐8 rather than caspase‐9 suggests that the actions of BMPs are precisely controlled by BMPER.

Different consequences of BMP‐Smad activation depend on functional Smads isoforms and the interaction between Smads with other transcription factors in a particular pathophysiological status. There are numerous contradict reports on the roles of Smad1, Smad2, and Smad3 in ischemic brain injury.^37^, ^38^, ^39^, ^40^, ^41^ In our work, we found that BMPER only activated Smad3 but not Smad1 or Smad2. Moreover, BMPER increased phosphorylation of Akt and Nrf2. These two molecules are downstream factors of Smad3 in myocardial infarction.^42^, ^43^ Akt can directly interact with Smad3 to regulate the sensitivity to TGF‐β‐induced apoptosis.^44^ Our data suggest that Smad3 may be a preferable target of BMPER upon ischemic stress. The activation of Smad3 by BMPER further leads to an activation of Akt‐Nrf2 signaling pathway, which is known to be involved in neuroprotective action of many compounds/factors.^45^, ^46^

One limitation of this study is that we did not examine whether recombinant BMPER could enter the brain parenchyma. It is known that the blood‐brain barrier (BBB) prevents majority of large molecules in the blood from entering the brain. The difficulty of delivering therapeutic agents to specific brain regions represents a major challenge to treat brain disorders.^47^ Nevertheless, previous work in animal models of stroke by Huang et al. has identified that there is a biphasic leakage of BBB, with an early opening within hours after hypoxia/ischemia followed by a refractory phase and a second opening the next day.^48^ Moreover, Liu and Su have noted that BBB is not a barrier in the development of new drugs for ischemic stroke since the permeability of BBB increases and allows many “impermeable” drugs to enter the brain. In addition, many novel protective agents produce beneficial effects via peripheral or systemic actions.^49^ In the present study, we tested the effect of BMPER on a neuron OGD/R model and found it to be protective, indicating that BMPER might act in a CNS‐related manner. Whether or how BMPER plays a role in the peripheral system needs further exploration.

Another limitation of this study is that we did not use female animals to test the neuroprotection of BMPER. Biological sex influences many variables in stroke or cerebral ischemia, including general health status, cerebrovascular anatomy and function, risk factors, and therapeutic response.^50^ Moreover, cerebral ischemia activates a variety of inflammatory cascades, which are also highly sex‐dependent.^51^ This difference in brain injury and post‐injury inflammation would certainly contribute to the differences in post‐stroke repair.^52^ Due to sex difference, the neural plasticity in human, females achieved maximum body growth approximately 2 years earlier than males, with a correspondingly earlier stabilization of brain metabolism to adult levels.^53^ As BMP9 is increased by estrogens in brown adipose tissue of mice^54^ and BMPER is a member of BMPs family, whether BMPER would be affected by sex is unknown. Moreover, Zhao et al. reported a higher proportion of small infarcts in C57BL/6J strain mouse, which was largely responsible for sex‐dependent differences.^55^ Since C57BL/6J mouse strain was applied in our study, whether BMPER benefits ischemic brain injury both in male and female is an intriguing question meriting further investigation in the near future.

## 5 CONCLUSIONS

Our study indicates that BMPER is a novel neuroprotective hormone. It alleviates cell death and neuroinflammation to limit ischemic brain injury via the activation of Smad3/Akt/Nrf2 signaling pathway. These findings may provide a potential therapeutic strategy for stroke in the future.

## CONFLICT OF INTEREST

The authors claim that there are no conflicts of interest.

## AUTHOR'S CONTRIBUTIONs

Dr. Peng Ding and Dr. Wei Chen contributed to designing the study, recruiting patients, performing the experiments, and writing the draft. Dr. Xiaodi Yan and Dr. Jinxiang Zhang contributed to analyzing the results and discussing the work. Dr. Cheng Li made substantial contribution to the process of major revision and performed the data presentation. Dr. Guangming Zhang and Dr. Yongqiang Wang contributed to supervising the work and revising the manuscript. Dr. Yonghua Li contributed to designing the study, providing resources, performing the experiments, and revising the manuscript.

## Supporting information

Fig S1

## Data Availability

The datasets generated and/or analyzed during the current study are available from the corresponding authors on reasonable request.
