# Supplementary figures and images for: BMPER alleviates ischemic brain injury by protecting neurons and inhibiting neuroinflammation via Smad3‐Akt‐Nrf2 pathway
Source: CNS Neurosci Ther. 2021 Dec 14;28(4):593–607. doi: 10.1111/cns.13782 (PMC8928915; doi:10.1111/cns.13782)

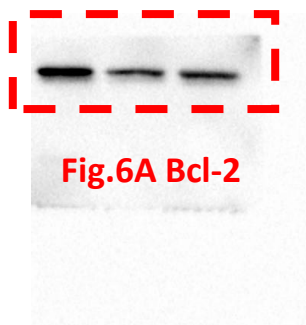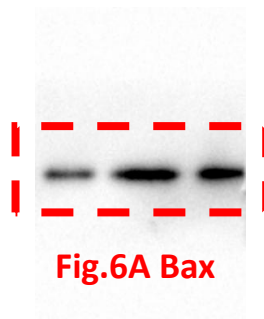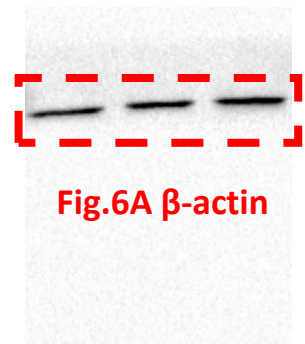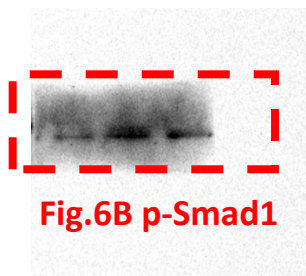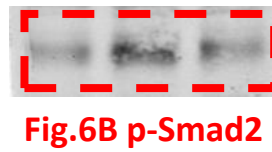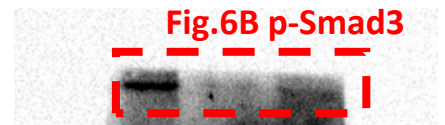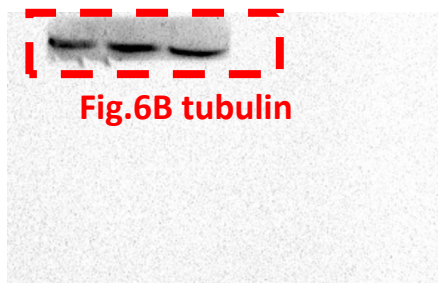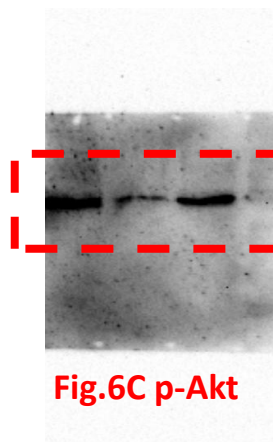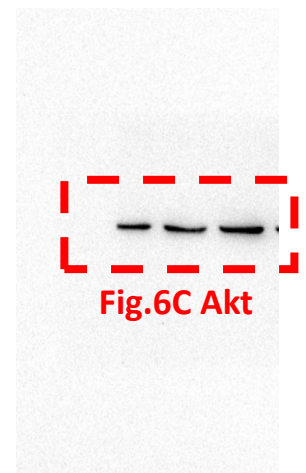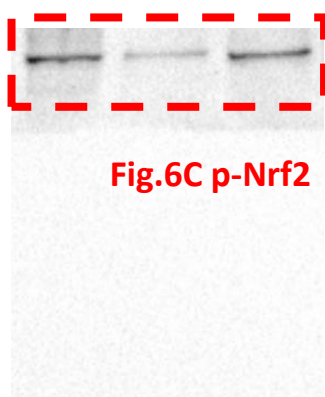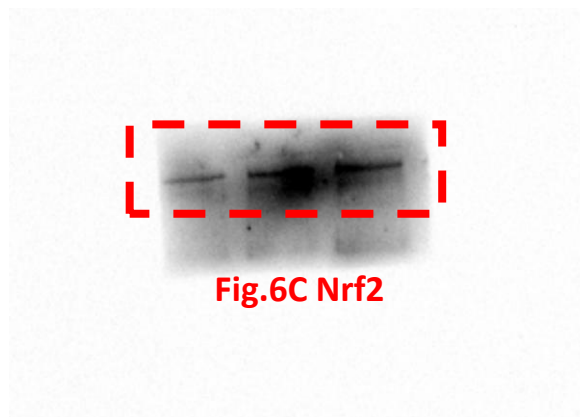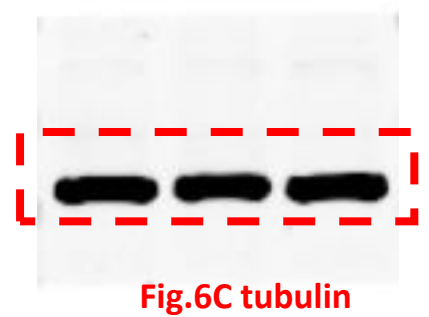

Supplement: Supplementary file 1 — Fig S1 [file CNS-28-593-s001.pdf]
